# Effects of antidromic and orthodromic activation of STN afferent axons during DBS in Parkinson's disease: a simulation study

**DOI:** 10.3389/fncom.2014.00032

**Published:** 2014-03-19

**Authors:** Guiyeom Kang, Madeleine M. Lowery

**Affiliations:** UCD School of Electrical, Electronic and Communications Engineering, University College DublinDublin, Ireland

**Keywords:** deep brain stimulation, antidromic, Parkinson's disease, beta band oscillations, computational modeling

## Abstract

Recent studies suggest that subthalamic nucleus (STN)-Deep Brain Stimulation (DBS) may exert at least part of its therapeutic effect through the antidromic suppression of pathological oscillations in the cortex in 6-OHDA treated rats and in parkinsonian patients. STN-DBS may also activate STN neurons by initiating action potential propagation in the orthodromic direction, similarly resulting in suppression of pathological oscillations in the STN. While experimental studies have provided strong evidence in support of antidromic stimulation of cortical neurons, it is difficult to separate relative contributions of antidromic and orthodromic effects of STN-DBS. The aim of this computational study was to examine the effects of antidromic and orthodromic activation on neural firing patterns and beta-band (13-30 Hz) oscillations in the STN and cortex during DBS of STN afferent axons projecting from the cortex. High frequency antidromic stimulation alone effectively suppressed simulated beta activity in both the cortex and STN-globus pallidus externa (GPe) network. High frequency orthodromic stimulation similarly suppressed beta activity within the STN and GPe through the direct stimulation of STN neurons driven by DBS at the same frequency as the stimulus. The combined effect of both antidromic and orthodromic stimulation modulated cortical activity antidromically while simultaneously orthodromically driving STN neurons. While high frequency DBS reduced STN beta-band power, low frequency stimulation resulted in resonant effects, increasing beta-band activity, consistent with previous experimental observations. The simulation results indicate effective suppression of simulated oscillatory activity through both antidromic stimulation of cortical neurons and direct orthodromic stimulation of STN neurons. The results of the study agree with experimental recordings of STN and cortical neurons in rats and support the therapeutic potential of stimulation of cortical neurons.

## 1. Introduction

Deep brain stimulation (DBS) is an established surgical therapy for treating the symptoms of medically refractory Parkinson's disease. The mechanisms by which DBS exerts its therapeutic influence, at both the cellular and system level, however, remain unclear. Excessively synchronized neural activity throughout the cortico-basal ganglia network is a characteristic hallmark of Parkinson's disease (Eusebio et al., 2008). Increased oscillatory activity in the beta frequency range (13–30 Hz) has been observed in the subthalamic nucleus (STN) and the striatum of MPTP-treated monkeys (Bergman et al., 1994; Raz et al., 1996), and in the STN, globus pallidus interna (GPi) and cortex of patients with Parkinson's disease (Brown et al., 2001; Kühn et al., 2005; Litvak et al., 2011). Beta-band oscillations in the local field potential (LFP) recorded from the STN are correlated with the symptoms of bradykinesia and rigidity in Parkinson's disease (Kühn et al., 2008; Bronte-Stewart et al., 2009). Non-linear signal analysis techniques have also revealed a causal relationship between oscillations in the 4–8 Hz range and parkinsonian tremor (Tass et al., 2010). It has been shown that beta-band power in the STN is largely attenuated during and immediately following STN-DBS (Kühn et al., 2008; Bronte-Stewart et al., 2009; Eusebio et al., 2011) and abolished following administration of levodopa (Kühn et al., 2006), supporting the hypothesis that DBS exerts at least part of its therapeutic influence by disrupting synchronous oscillations among neural circuits in the brain. The exact mechanisms by which DBS suppresses abnormal neural activity in the cortico-basal ganglia network, however, remain unknown.

There are several potential target sites at which STN-DBS may modulate neuronal behavior. These include local axons of passage, the STN soma and afferent fibers descending from the cortex to the STN through what is known as the “hyperdirect” pathway (McIntyre et al., 2004; Miocinovic et al., 2006; McIntyre and Hahn, 2010). In addition to orthodromic effects of stimulation, in the direction from the dendrites to axon terminal, a number of studies have provided strong evidence that STN-DBS may modulate cortical activity through antidromic stimulation, in the direction from axon terminal to the soma, resulting in suppression of excessive oscillatory activity in both cortical and STN neurons. In human subjects recording of evoked potentials in the motor cortex suggested that antidromic activation of STN stimulation may activate and modulate the activity of cortical neurons (Ashby et al., 2001; Baker et al., 2002; MacKinnon et al., 2005). Observations in 6-hydroxydopamine (6-OHDA) lesioned rats and rats administered with a combined D1/D2 antagonist have also shown that STN-DBS results in the direct antidromic activation of cortical neurons (Li et al., 2007; Dejean et al., 2009) and furthermore that it suppresses pathological beta oscillations in the cortex as shown in recorded single-unit activities from populations of the cortical neurons in layer V of M1 (Li et al., 2012). Recent experiments using optogenetic technology suggest that STN afferent axons originating from the cortex may be the main target of DBS, as selective stimulation of afferent axons to the STN resulted in restoration of motor behavior close to normal in 6-OHDA lesioned rat and was more effective than direct STN stimulation (Gradinaru et al., 2009). Further evidence of antidromic activation is provided by observations of increased neural activation in the cortex and STN of MPTP treated non-human primates during high frequency STN-DBS, attributed to a combination of antidromic and orthodromic activation throughout the cortico-basal ganglia network (Santaniello et al., 2012). Whitmer et al. (2012) observed that suppression of beta synchronization was greatest at the estimated origin of the hyperdirect pathway between the motor cortex and the STN. Their results further support the idea that suppression of beta activity within the motor cortex in patients with Parkinson's disease may be due to antidromic activation. However, in that study it was not possible to reliably separate the relative contributions of antidromic and orthodromic effects due to the high frequency nature of the stimulation (Whitmer et al., 2012).

While the above studies suggest that antidromic activation may play an important role in the manner in which STN-DBS mediates its therapeutic effects, other studies have indicated that antidromic activation occurs in only a subset of afferent neurons (Canteras et al., 1990) and that the probability of antidromic firing is low (Chomiak and Hu, 2007; Hammond et al., 2008). Consequently, it has also been argued that the role played by antidromic activation in suppressing pathological oscillations in the beta frequency range may be relatively small when compared with direct orthodromic effects (Chomiak and Hu, 2007).

It is difficult to separate the effects of orthodromic and antidromic stimulation arising as a result of STN-DBS using *in vitro* or *in vivo* methods. Computational or *in silico* modeling provides an alternative means by which to examine each of these effects in isolation and provides access to parameters not accessible experimentally. Computational modeling has indicated that antidromic activation of primary axons and axon collaterals using STN-DBS results in faithful propagation of action potentials across a wide range of axonal geometric conditions (Grill et al., 2008). The aim of the present study was thus to examine the relative effects of antidromic and orthodromic activation of STN afferent axons originating from the cortex during STN-DBS. Using a computational model of the cortico-STN-GPe network, the effects of antidromic and orthodromic stimulation were examined, both in combination with overlapping effects, as well as in isolation, by allowing the contribution of each effect to be separately excluded. A new computational model of the cortico-STN-GPe network was developed incorporating DBS of STN afferent axons descending from the cortex through the hyperdirect pathway. Firing activity in both cortical and STN neurons, including the effect of STN-DBS on pathological beta-band oscillations, was examined as the stimulation frequency was systemically varied and the effects of low frequency STN-DBS and high frequency STN-DBS were compared. The model presented here is to first to simulate the network effects of DBS of STN afferent axons originating in the cortex and to address the effects of antidromic and orthodromic activation of STN-DBS on cortical and STN neurons.

## 2. Materials and methods

**Figure 1:**
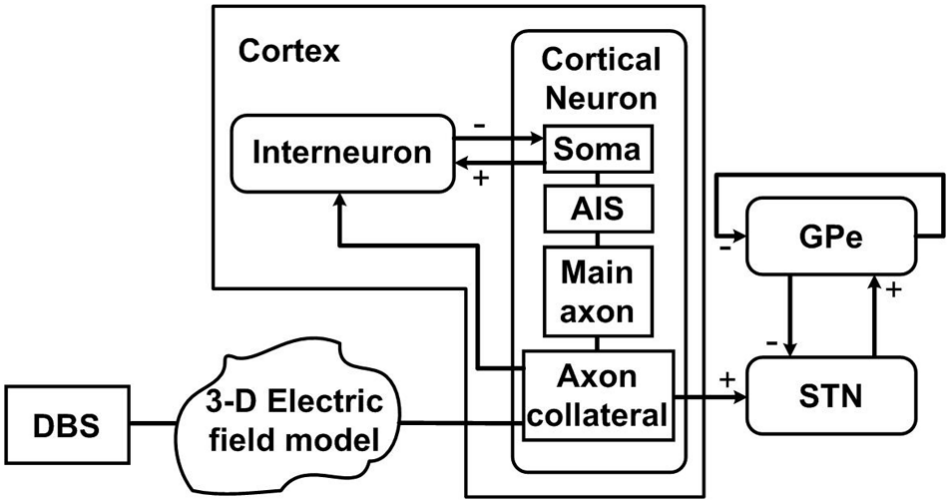
**Schematic diagram of the model, illustrating connections between the cortex, STN and GPe, and DBS applied extracellularly along the cortical axon collateral branches**. Each cortical neuron has four compartments including the soma, axon initial segment (AIS), main axon, and axon collateral. Each nucleus consists of 100 neurons. “+” signs indicate excitatory pathways while “−” signs represent inhibitory pathways.

DBS was applied extracellularly to a model of the cortico-basal ganglia network which was comprised of groups of interconnected neurons within the cerebral cortex (cortex), STN and GPe. The model used in this study is based on the network model developed by Kang and Lowery (2013), with simulation of synaptic coupling between neurons and the parameters for the STN and GPe neurons as described in Kang and Lowery (2013). In addition, the model has several adaptations to capture a more physiological representation of the extracellular application of DBS and two classes of cortical neurons. The simulated cortex included excitatory pyramidal neurons (cortical neurons) and inhibitory interneurons (interneurons). Each multi-compartment cortical neuron consisted of four parts, a soma, axon initial segment (AIS), main axon and axon collateral, while the neurons within the STN, GPe and interneurons were implemented as single-compartment models. For the purposes of this simulation study, it was assumed that the dominant mechanism through which STN-DBS achieves its therapeutic effect is through the activation of afferent fibers descending from the cortex to the STN through the hyperdirect pathway following the conclusions of the recent optogenetic study by Gradinaru et al. (2009). A schematic diagram of the model is presented in Figure 1.

The extracellular potential in the surrounding white matter tissue during DBS was estimated by modeling the applied stimulus as a point current source under the assumption of quasistatic conditions (Plonsey and Heppner, 1967; Zhang and Grill, 2010). The stimulus current, *I*_DBS_ was delivered to a homogeneous extracellular medium of conductivity σ. The extracellular potential, *V*_*e*_ at each point *i* located a distance (*x*_*i*_, *y_i_*, *z_i_*) from the point source positioned at the origin was calculated as:

(1) $$V_{e}=\frac{I_{\text{DBS}}}{4\text{​}\pi \text{​}\sigma \sqrt{x_{i}^{2}+y_{i}^{2}+z_{i}^{2}}}$$

The DBS current, *I*_DBS_ was simulated as a series of periodic rectangular current pulses of variable amplitude, frequency, and duration.

(2) $$I_{\text{DBS}}=i_{D}H\left\{\text{sin}\left(\frac{2\pi t}{\rho_{D}}\right)\right\}\left[1-H\left\{\text{sin}\left(\frac{2\pi (t+\delta_{D})}{\rho_{D}}\right)\right\}\right]$$

where *H* is the Heaviside step function, *H*(*x*) = 0 for *x* < 0, and *H*(*x*) = 1 for *x* ≥ 0, ρ_*D*_ is the interstimulus interval, *I*_*D*_ is the maximum pulse amplitude, and δ_*D*_ is the duration of the positive phase of the stimulus pulse.

### 2.1. Cortical neurons

The cortical neuron model consisted of a soma, AIS, main axon and axon collateral situated in 3-dimensional space. The cortical soma model is based on regular spiking cortical neurons developed by Pospischil et al. (2008). It represents a simplified single-compartment Hodgkin–Huxley type model which includes intrinsic properties of voltage dependent conductances described by a series of differential equations for cortical neurons. The membrane potential of the cortical soma of neuron, *i*, *V*_*m*_soma_*i*___ is described by

(3) $$\frac{dV_{m_{{\text{soma}}_{i}}}}{dt}=\frac{1}{C_{m_{{\text{soma}}_{i}}}}\left(-I_{Na_{{\text{soma}}_{i}}}-I_{Kd_{{\text{soma}}_{i}}}-I_{M_{{\text{soma}}_{i}}}-I_{{\text{leak}}_{{\text{soma}}_{i}}}\right)$$

where *C*_*m*_soma_*i*___ is the membrane capacitance, *V*_*m*_soma_*i*___ is the membrane potential, *I*_*Na*_soma_*i*___ is the sodium current, *I*_*Kd*_soma_*i*___ is the potassium current and *I*_*M*_soma_*i*___ is a slow voltage-dependent potassium current responsible for spike-frequency adaptation. *I*_leak_soma_*i*___ indicates a leak current. Details of the parameter values for the cortical neurons are as presented in Pospischil et al. (2008).

The model used to simulate the cortical axon including the AIS, main axon and axon collateral is adopted from the results of previous modeling and experimental findings (Foust et al., 2011). Similar to the equation for the cortical soma, the membrane potentials of the AIS, main axon and axon collateral of neuron, *i*, *V*_*m*_axon_*i*___ are each described by

(4) $$\frac{dV_{m_{{\text{axon}}_{i}}}}{dt}=\frac{1}{C_{m_{{\text{axon}}_{i}}}}\left(-I_{Na_{{\text{axon}}_{i}}^{+}}-I_{K_{{\text{axon}}_{i}}^{+}}-I_{Kd_{{\text{axon}}_{i}}}-I_{{\text{leak}}_{{\text{axon}}_{i}}}\right)$$

where *C*_*m*_axon_*i*___ is the membrane capacitance, *V*_*m*_axon_*i*___ is the membrane potential, *I*_*Na*^+^_axon_*i*___ is the sodium current, *I*_*K*^+^_axon_*i*___ is the delayed rectifier potassium current and *I*_*Kd*_axon_*i*___ is the rapidly activating, slowly inactivating *K*^+^ current. *I*_leak_axon__*i*__ represents the leakage current. Sodium conductances, *g_Na_i__* were chosen to be 90, 200, 533, and 500 pS/μm^2^ in the AIS, nodes, collaterals and internodes, respectively. Similarly, 120, 30, 10, and 50 pS/μm^2^ were the potassium conductances, *g*_*K*^+^_*i*__ in the AIS, nodes, collaterals and internodes, respectively. *g*_*Kd*_*i*__ was distributed as 6, 3, 10, and 0 pS/μm^2^ in the AIS, nodes, collaterals and internodes. *g*_leak_*i*__ was set to 0.23 pS/μm^2^ in all axon compartments. Details of the equations and other parameter values for the cortical axon are as presented in Foust et al. (2011).

**Figure 2:**
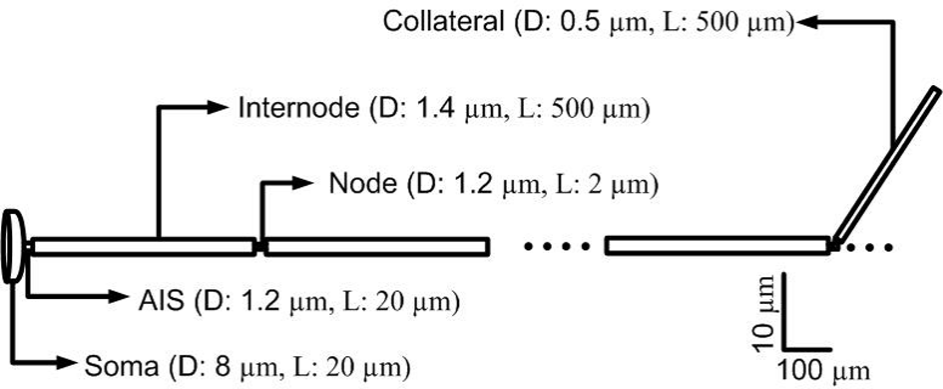
**Morphology of a single simulated cortical neuron**. The model consisted of a soma, axon initial segment (AIS), myelinated axon with 10 nodes separated by 10 internodal spacings of 500 μm. An unmyelinated axon collateral is attached to the last node. (D, diameter; L, Length).

**Figure 3:**
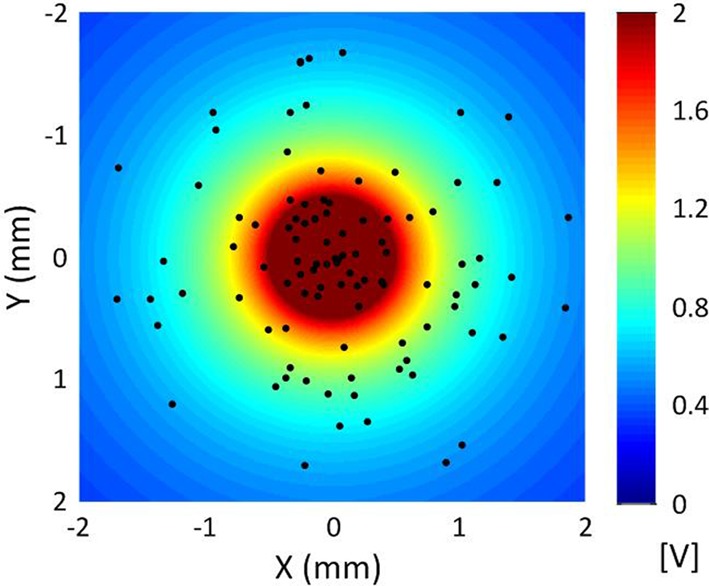
**Distribution of cortical axon collaterals in the vicinity of a point electrode located at the origin for a sample simulation**. The axon collaterals are orientated with the axons parallel to one another in the direction into the page. The extracellular potential generated by the point source electrode is also indicated.

In terms of geometry, the single-compartment soma of each cortical neuron model was assumed to have length 35 μm and diameter 25 μm (Yu et al., 2008). The soma connected to the AIS which had a length of 20 μm and diameter of 1.2 μm, connected in turn to a myelinated axon comprised of 10 nodes each of length of 2 μm and diameter of 0.5 μm separated by 10 internodes of length 500 μm and diameter 1.4 μm (Yu et al., 2008; Foust et al., 2011). An unmyelinated collateral axon 500 μm long and 0.5 μm in diameter was attached to the last node (Foust et al., 2011). The model morphology is presented in Figure 2. The cortical axon collaterals were randomly located throughout the STN and each collateral was synaptically connected to the 10 nearest STN neurons as estimated using the Euclidean distance, Figure 3.

Experimental studies indicate that STN-DBS is likely to activate many local axon collaterals emitted from small and medium sized corticofugal axons originating from the frontal cortex layer V neurons and connecting to the cortex (Li et al., 2007; Kita and Kita, 2012). Therefore, in the model DBS was applied to the cortical axon collaterals and the extracellular potential generated by the point current source representing the electrode was coupled to the multi-compartment model of axon collateral, *i*, in the form described in Rattay (1986).

(5) $$C_{m_{i}}\frac{V_{n_{i}}}{dt}=G_{a_{i}}\left(V_{n-1_{i}}-2V_{n_{i}}+V_{e,n-1_{i}}-2V_{e,n_{i}}+V_{e,n+1_{i}}\right)-I_{c,n_{i}}$$

Here, *C_m_i__* is the membrane capacitance, *G_a_i__* is axon conductance, *V_e,n_i__* is extracellular potential of the *n*th compartment and *I_c,n_i__* is the ionic current passing through the *n*th compartment. *V_n_i__* is the reduced membrane voltage defined as *V_n_i__* = *V_i,n_* − *V_e,n_* + *V*_rest_, where *V_i, n_* is internal potential and *V*_rest_ is internal resting potential.

### 2.2. Interneurons

One of the major classes of neurons in the cortex are the interneurons which are reciprocally connected to the cortical pyramidal neurons. It has been suggested that interneurons may be stimulated indirectly by STN-DBS through the cortical afferents and as a consequence that the induced activation of interneurons disrupts neural firing of the cortical neurons (Li et al., 2007, 2012; Fraix et al., 2008). The model interneurons here are stimulated directly to represent their stimulation through the cortical afferents. In this study, interneurons were represented using the model developed by Izhikevich (2003). It is a relatively simple, biologically plausible spiking model which reduces the computational burden relative to the full Hodgkin–Huxley type equations. The simplified model was chosen to be sufficient here as its primary role is to act as a source for the inhibitory input to cortical neurons and its firing threshold produces synaptic input consistent with other synaptic inputs. Although the other neurons are continuous with respect to the membrane potential and the interneurons are effectively threshold crossing models which are discontinuously reset, once an action potential is generated in either model it leads to the generation of a synaptic EPSP/IPSP in the same manner in all models. The mathematical equation describing the membrane potential of interneuron *i*, *V*_*m*_IN_*i*___ is given by a series of ordinary differential equations of the form

(6) $$\begin{array}{l} \text{​}\frac{dV_{m_{{\text{IN}}_{i}}}}{dt}=0.04V_{m_{{\text{IN}}_{i}}}^{2}+5V_{m_{{\text{IN}}_{i}}}+140-u_{i}+I_{\alpha_{i}\to \beta_{i}}\\ \;\frac{du_{i}}{dt}=a\left(bV_{m_{{\text{IN}}_{i}}}-u_{i}\right) \end{array}$$

with the auxiliary after-spike resetting as follows

(7) $$\text{if }V_{m_{{\text{IN}}_{i}}}\geq 30mV,\text{then}\{\begin{array}{ll} V_{m_{{\text{IN}}_{i}}} & \to c\\ u_{i} & \to u_{i}+d \end{array}$$

Here, *V*_*m*_IN_*i*___ represents the membrane potential and *u*_*i*_ represents a membrane recovery variable related to activation of *K*^+^ ionic currents and inactivation of *Na*^+^ ionic currents that provides negative feedback to *V*_*m*_IN_*i*___. The parameter *a* describes the time scale of *u*_*i*_ and parameter *b* describes the sensitivity of the recovery variable *u*. The parameter *c* describes the after-spike reset value of the membrane potential of −65 mV. Finally, the parameter *d* describes after-spike reset of the recovery variable *u*_*i*_.

### 2.3. STN and GPe neurons

The STN neuron was simulated using the model introduced by Otsuka et al. (2004). This model incorporates STN plateau potentials which have been shown to play a critical role in the generation of bursting activity in Parkinson's disease (Beurrier et al., 1999). The membrane potential of STN neuron *i*, *V*_*m*_STN_*i*___ is described by

(8) $$C_{m_{{\text{STN}}_{i}}}\frac{dv_{m_{{\text{STN}}_{i}}}}{dt}=-I_{Na_{{\text{STN}}_{i}}}-I_{K_{{\text{STN}}_{i}}}-I_{A_{{\text{STN}}_{i}}}-I_{L_{{\text{STN}}_{i}}}$$

(9) $$-I_{T_{{\text{STN}}_{i}}}-I_{Ca-K_{{\text{STN}}_{i}}}-I_{{\text{leak}}_{{\text{STN}}_{i}}}$$

where *C*_*m*_STN_*i*___ is the membrane capacitance and the ionic current channels included are the sodium, *I*_*Na*_STN_*i*___, high activation threshold potassium *I*_*K*_STN_*i*___, low activation threshold potassium *I*_*A*_STN_*i*___, long-lasting calcium *I*_*L*_STN_*i*___, low-threshold calcium *I*_*T*_STN_*i*___, calcium-activated potassium *I*_*Ca*−*K*_STN_*i*___, and leakage *I*_leak_STN_*i*___ currents.

The model used to simulate the GPe neuron was adapted from that presented in Rubin and Terman (2004), with the membrane potential for neuron, *i* described as follows

(10) $$\frac{V_{m_{i}}}{dt}=-\frac{1}{C_{m_{i}}}\left(\sum_{j}I_{j_{i}}+I_{{\text{syn}}_{i}}\right)$$

where *C_m_i__* is membrane capacitance and *V_m_i__* is membrane potential. *I_j_i__* indicates the individual ionic currents required to generate the membrane potential for each neuron and *I*_syn_*i*__ represents the synaptic current. Details of parameter values and ionic currents are as given in Kang and Lowery (2013).

### 2.4. Synaptic connectivity

One hundred neurons were simulated in each population of neurons in the model (STN, GPe, cortical neurons and interneurons). Preliminary studies using the model indicated that this number is sufficient to capture the network effects of interest while maintaining a reasonable level of computational efficiency. For simplicity the same number of neurons was assumed in each nucleus was used. In reality, however, the number of neurons varies across the different nuclei. In particular, the ratio of cortical neurons to interneurons is about approximately 200:20 (McCarthy et al., 2008). The total number of neurons in the model is substantially greater than those used in previous network models. For example, the model of Terman et al. (Terman et al., 2002; Rubin and Terman, 2004) used 16 neurons to simulate each of the STN, GPe, and globus pallidus interna (GPi). While the direction of connections between the different nuclei is well established, it is more difficult to obtain estimates of the exact numbers of neurons to which each neuron is connected. For this reason we implemented a simple scheme that enabled current knowledge on the direction of connectivity between the nuclei to be incorporated, with each neuron receiving excitatory input in the form of excitatory post synaptic currents (EPSCs) or inhibitory input in the form of inhibitory post synaptic currents (IPSCs) from 1 or 2 other neurons. The connectivity from cortical neurons to STN neurons assumed that the STN receives substantial direct cortical input (Albin et al., 1989). To represent this connection, each STN neuron received excitatory input from approximately 10 cortical neurons randomly chosen in the model. Each STN neuron received inhibitory input from the single GPe neuron located closest to it (Smith et al., 1990; Fujimoto and Kita, 1993) and provided an excitatory input to the two closest GPe neurons (Nakanishi et al., 1987). To capture self-inhibition within the GPe (Kita and Kitai, 1994), each GPe neuron additionally received inhibitory input from the two closest GPe neurons. An all-to-all connection scheme was used for the synaptic connectivity between cortical neurons and interneurons as used in a previous model of cortical network activity (McCarthy et al., 2008). At each synaptic connection, the synaptic current *I*_α_*i*_→β_*i*__ is given by

(11) $$I_{\alpha_{i}\to \beta_{i}}=g_{\alpha_{i}\to \beta_{i}}(v_{\beta_{i}}-v_{\alpha_{i}\to \beta_{i}})\sum_{j=1}^{n}s_{j}$$

where *g*_α_*i*_→ β_*i*__ indicates the synaptic gain and *v*_α_*i*_→ β_*i*__ is the synaptic reversal potential of the synaptic current. The sum is taken over all presynaptic neurons connected to each neuron. Each synaptic variable *s*_*j*_ was simulated using an alpha function to describe a conductance that has a rising phase as follows

(12) $$s_{j}=\frac{t-t_{0}}{\tau }e^{1-\frac{t-t_{0}}{\tau }}$$

Here, a single time constant τ is the time course of the rising phase. The rise time for both EPSCs from the cortex to the STN and IPSCs from the GPe to STN was assumed to be 0.8 ms (Baufreton et al., 2005). Rise times of 0.86 and 0.2 ms were chosen for IPSCs from the GPe to GPe and EPSCs to the STN from the GPe, respectively (Hanson et al., 2004; Sims et al., 2008). Generic values of 0.5 ms and 0.93 ms for EPSCs from the cortical neurons to interneurons and IPSCs from the interneurons to the cortical neuron, respectively, were used (Jaeger, 2003).

Gaussian white noise was added to the membrane potential of each cortical, STN, GPe neuron and interneuron. For each neuron, additive noise was assumed with a mean of 0 and standard deviation of 0.001 μA/cm^2^.

### 2.5. Simulation details

Experimental evidence indicates that beta-band oscillatory activity of neural populations in the cortex precedes activity in the STN with which it is coherent under dopamine depleted conditions, suggesting that cortical inputs in this frequency range may drive STN beta-band activity in Parkinson's disease (Brown and Williams, 2005; Sharott et al., 2005a,b). Following this hypothesis, recent computational models have simulated the generation of beta activity in the STN as a result of strong cortical inputs under simulated parkinsonian conditions (Kang and Lowery, 2009; Hahn and McIntyre, 2010; Holgado et al., 2010; Kang and Lowery, 2013). To simulate the increased beta-band oscillatory activity observed in Parkinson's disease, beta-band oscillatory firing of the cortical neurons was simulated and the synaptic gain between cortex and STN was increased, resulting in synchronous oscillatory activity of STN and GPe neurons. Self-inhibition between GPe neurons was reduced and the synaptic gain between the GPe-STN and the STN-GPe was increased consistent with previous studies (Terman et al., 2002; Cruz et al., 2009).

**Figure 4:**
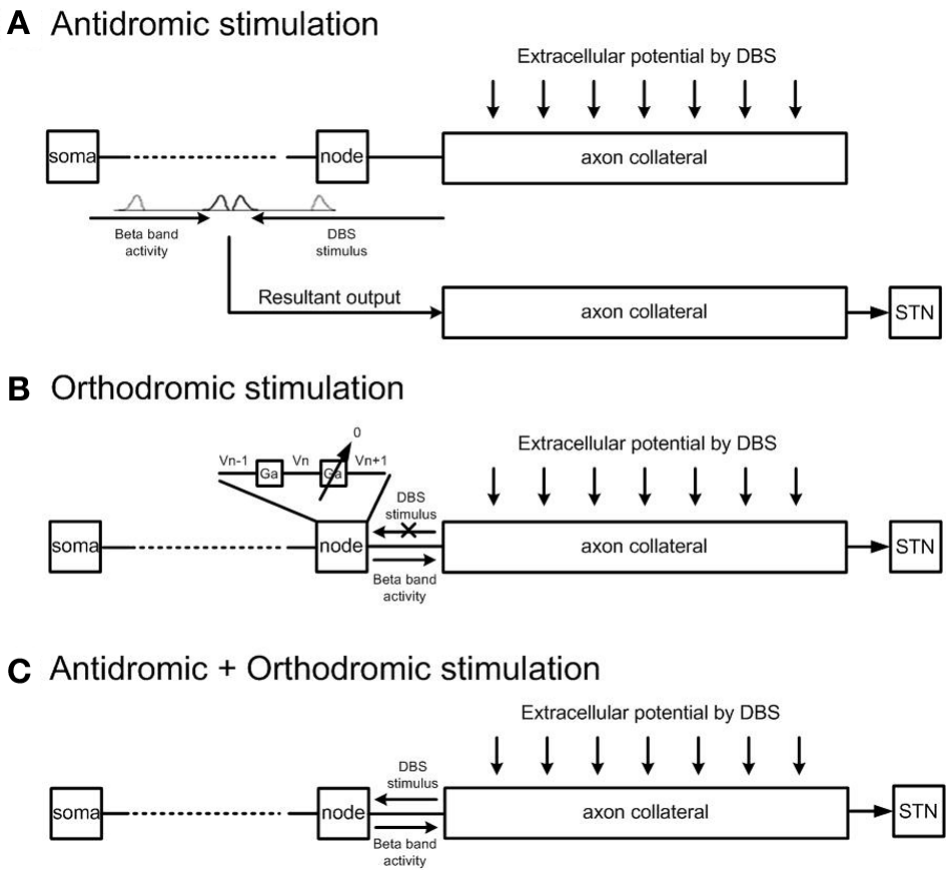
**Model architecture for antidromic and orthodromic activation of STN-DBS. (A)** Simulation of antidromic stimulation alone. Action potentials elicited in the axon collateral due to DBS propagate in the antidromic direction only. Action potentials generated within the cortex propagate orthodromically toward the STN. Action potential cancelation occurs on collision with antidromically propagating action potentials, while antidromically propagating action potentials reaching the cortex result in facilitation of cortical neurons. **(B)** Simulation of orthodromic activation alone. STN-DBS results in orthodromic propagation of action potentials along the axon collateral toward the STN. **(C)** In the case where both antidromic and orthodromic activation are permitted, DBS is injected to the cortical axon collateral and the resultant action potential travels in both directions along the axon collateral.

The effects of (1) antidromic, (2) orthodromic, and (3) simultaneous antidromic and orthodromic excitation of collaboratoral axons due to extracellular DBS were each examined. Definition of antidromic and orthodromic activation of STN-DBS used in the model was illustrated in Figure 4. To simulate the effect of antidromic activation in isolation, each axon collateral was represented by a pair of identical axon collaterals simulated in parallel. One axon collateral was influenced by the extracellular potential and was bi-directionally connected to the cortical axon but not to the STN, while the other axon collateral which was connected to the STN received the resultant output of the combined effects of cortical excitation and antidromic activation on the cortical neuron only. This resulted in the path of STN-DBS activated action potentials traveling in the orthodromic direction toward the axon terminal being blocked. Antidromically propagating action potentials, however, were allowed to propagate into the primary axon toward the soma. The cortical soma also received synaptic inputs from inhibitory interneurons in the cortex and the resulting cortical output propagated back toward the STN neurons.

To simulate the effect of pure orthodromic activation due to STN-DBS, action potentials traveling toward the cortical soma were blocked by setting the axon conductance of the last node connected to the axon collateral close to zero. This resulted in orthodromic activation of the axon during STN-DBS. As propagation was blocked in the direction toward the cortical soma only, action potentials generated within the cortex were allowed to reach the axon collaterals but no antidromic propagation was allowed. Simultaneous antidromic and orthodromic excitations were simulated by allowing bi-directional propagation of action potentials during STN-DBS as in the physiological situation.

Simulations were performed on a personal computer with Intel(R) Core(TM)2 CPU 6600, 2.39 GHz and 3 GB RAM using C++. The simulation was performed for a duration of 5 s with DBS applied for 1 s in five separate simulation experiments and the power of the sum of the membrane potentials of the populations of STN and cortical neurons in the frequency band of interest was examined. User developed programs using Welch's method in Matlab (An adaptive-step fourth order Runge–Kutta method was used to solve the differential equations with a time step of 0.01 ms.

## 3. Results

**Figure 5:**
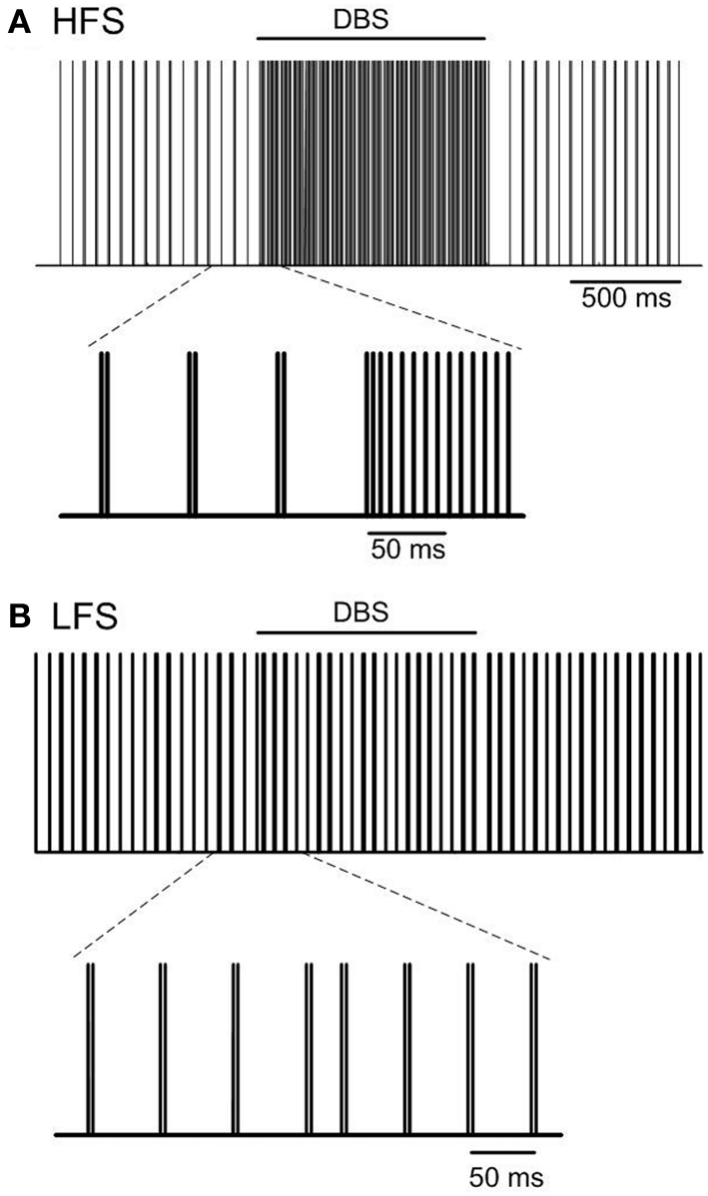
**Effect of DBS on periodic oscillatory activity in the beta frequency range in the STN**. Pulse trains representing the firing times of a representative STN neuron during high frequency (130 Hz) and low frequency (20 Hz) DBS with pulse amplitude and pulse duration of 2.5 mA and 60 µs are presented in **(A,B)**. (HFS, high frequency stimulation; LFS, low frequency stimulation).

**Figure 6:**
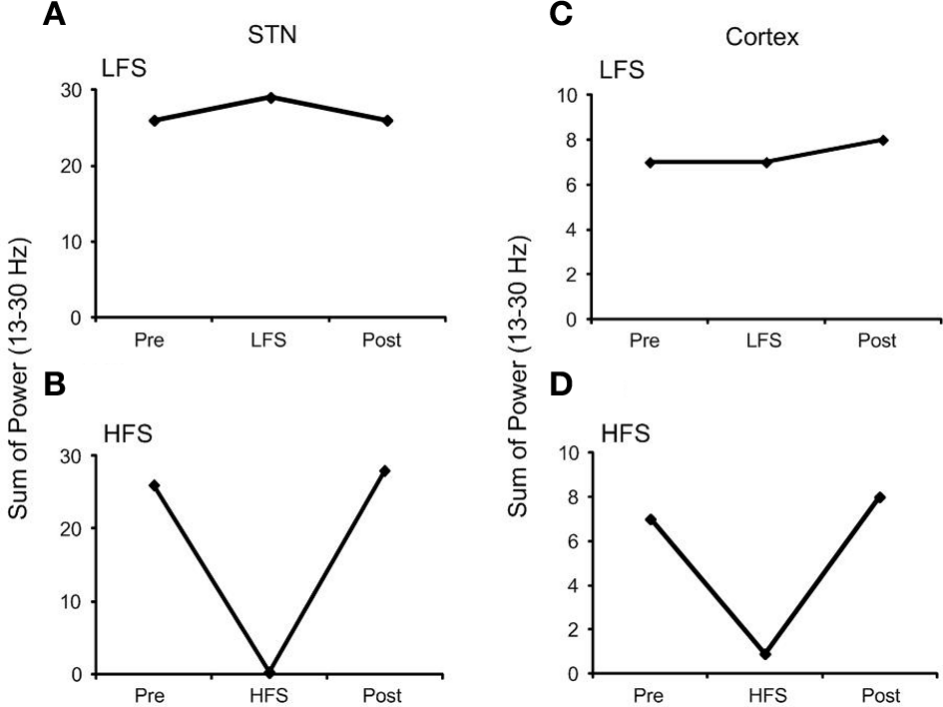
**Effect of DBS on the beta-band power of a representative STN and cortical neuron. (A)** Low frequency DBS increased beta-band power of the STN neuron while **(B)** high frequency DBS suppressed beta-band power. In the cortical neuron **(C)** low frequency DBS had no effect on beta-band power and **(D)** a similar reduction in beta-band power was observed when high frequency DBS was applied. (HFS, high frequency stimulation; LFS, low frequency stimulation).

The effect of high frequency (130 Hz) and low frequency (20 Hz) DBS on simulated beta frequency oscillations among the simulated STN neurons with both orthodromic and antidromic propagation facilitated was first examined. DBS with an amplitude of 2.5 mA and pulse duration of 60 µs for both conditions was applied, Figure 5. Consistent with previous experimental and modeling studies, high frequency DBS was observed to disrupt beta oscillatory activity in the STN neurons, with all neurons firing in response to each DBS input stimulus and periodic oscillatory activity of the STN neurons suppressed, Figure 5A. When the DBS frequency was reduced to 20 Hz, the firing pattern of the STN neurons was not affected, Figure 5B. The beta-band power of the membrane potential of the STN neurons decreased when high frequency DBS was applied, while a slight increase in beta-band power was observed when low frequency DBS at 20 Hz was applied, Figures 6A,B. Beta-band power in the cortical neurons was similarly reduced when high frequency DBS was applied, while low frequency DBS at 20 Hz had little effect on the power in the beta-band among the cortical neurons, Figures 6C,D.

### 3.1. Effects of STN-DBS on beta-band neural activity

#### 3.1.1. Separating antidromic and orthodromic effects of STN-DBS on neural firing in the STN

**Figure 7:**
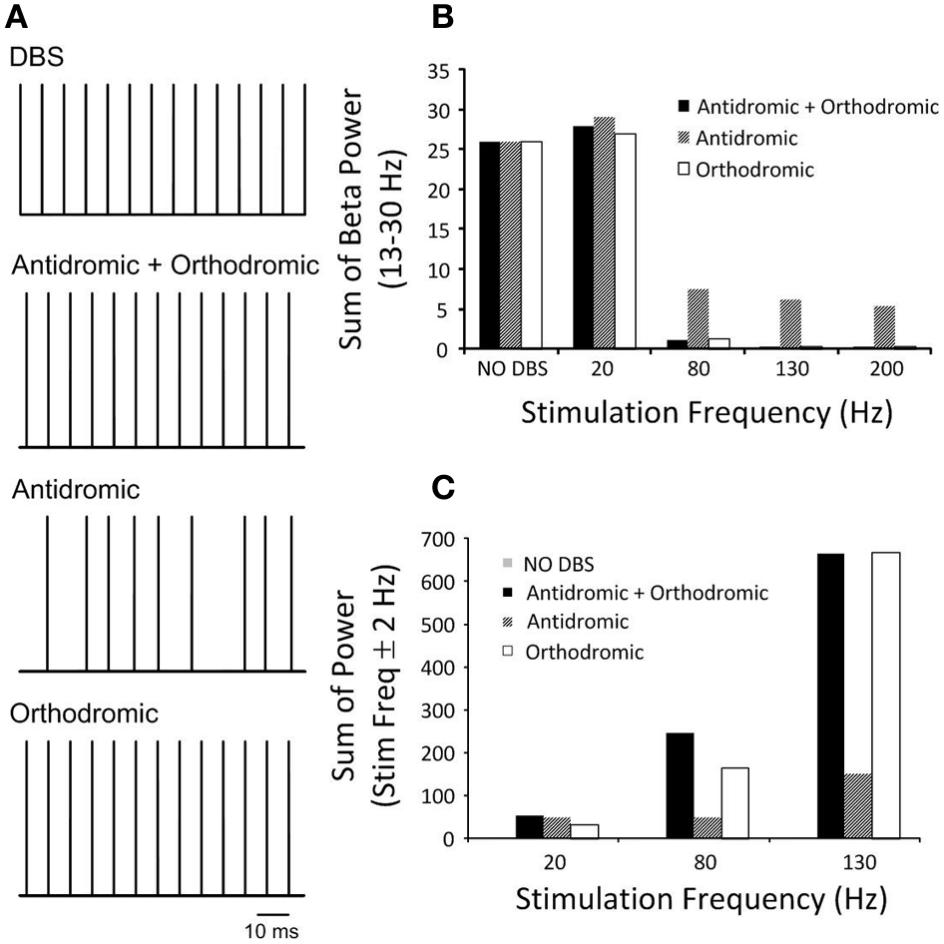
**(A)** Pulse trains represent the firing times of a representative STN neuron during DBS with an amplitude of 2.1 mA and pulse duration of 60 μs under the three simulated conditions. Combined antidromic and orthodromic activation and orthodromic activation alone during DBS produced neural firing in the STN driven by the stimulation pulse, while antidromic activation resulted in a low firing probability and random neural firing in the STN. **(B)** STN-DBS modulated neural firing of STN neuron and the effect of DBS frequency on the beta-band power of STN neurons is presented as the frequency of DBS was systemically varied for simulations conducted with antidromic, orthodromic and both antidromic and orthodromic effects of DBS included. **(C)** The sum of the STN power in the range ±2 Hz about the stimulation frequency is presented.

The effects of simultaneous antidromic and orthodromic activation of the cortical axon collaterals during DBS on the power spectrum of the STN neurons in the beta frequency range is shown in Figure 7 for a range of DBS frequencies.

The combined effect of antidromic and orthodromic activation resulted in stimulus-driven firing in the STN neurons, Figure 7A and a decrease in the beta-band power as the frequency of the DBS stimulus was increased, Figure 7B. In contrast, the sum of the STN power at the frequency of the applied stimulation increased as DBS frequency was increased, Figure 7C.

Firing of STN neurons in response to each DBS stimulus was also observed under the simulated condition of pure orthodromic activation, Figure 7A. A reduction in the beta-band power and increase in sum of the power of the DBS frequency range were also obtained as DBS frequency were systemically increased, Figures 7B,C.

In contrast to the results with combined orthodromic-antidromic activation and orthodromic activation alone, an irregular firing pattern was observed in the STN neurons under conditions of pure antidromic DBS activation, Figure 7A. As the frequency of the DBS stimulus was increased, the beta-band power decreased, Figure 7B. However, the suppression of beta-band activity during high frequency antidromic activation applied in isolation was less than that observed under either the combined effect of antidromic and orthodromic DBS or the application of orthodromic DBS alone. Similar to the other conditions, the sum of the power about the DBS frequency increased as DBS frequency was increased, Figure 7C, with the highest power observed during high frequency DBS. However, the amount by which the STN power at the stimulation frequency increased during high frequency DBS for antidromic activation was substantially lower when compared to that observed during the combined effect of antidromic and orthodromic activation or orthodromic activation alone.

**Figure 8:**
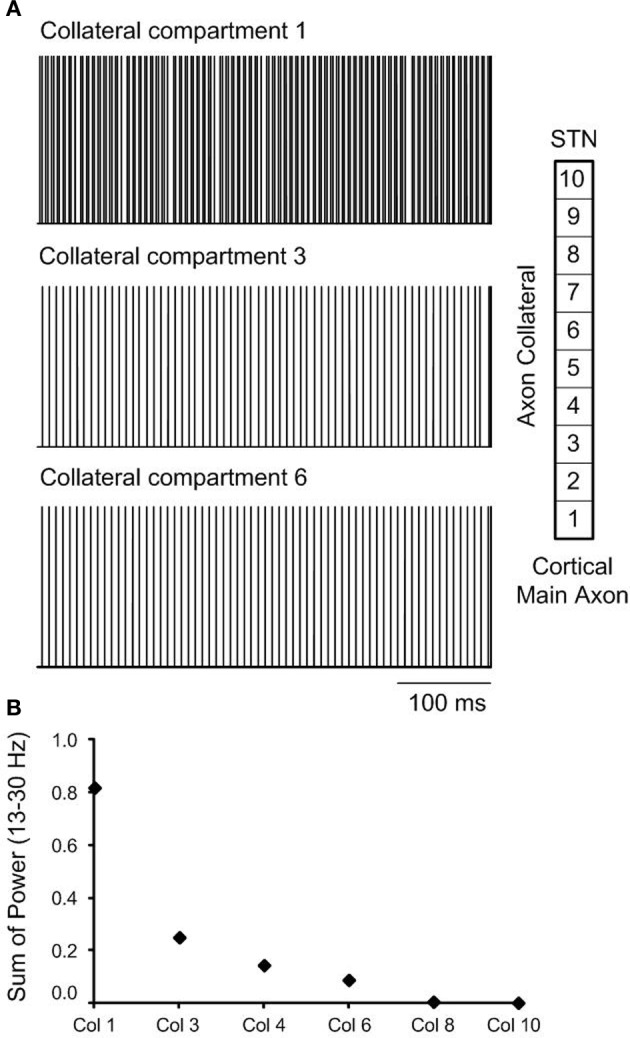
**(A)** The effect of orthodromic activation, only on neural firing in axon collateral compartments during high frequency DBS is presented. **(B)** Beta-band power in axon collateral compartments decreased as the action potentials propagated to toward the termination of the axon collateral.

As the effect of orthodromic activation during DBS resulted in a similar effect on neural firing in the STN to the combined effect of antidromic and orthodromic activation, the propagation of action potentials along the axon collaterals during DBS was examined to better understand the responses elicited by the different simulation conditions, Figure 8. In the case of orthodromic activation of DBS only, action potentials generated in the cortex propagated down to the point of branching where the axon collateral is attached to the main axon, without disturbance. Once action potentials entered the axon collateral, the beta-band activity decreased due to a dominant effect of DBS, resulting in the subsequent loss of action potentials originating in the cortex. Beta oscillatory activity was thus substantially suppressed in the axon compartment close to the axon terminal that was synaptically connected to the STN neuron, Figure 8A, and neural firing in response to each DBS stimulus was instead observed. The beta-band power in the various axon compartments decreased as action potentials propagated toward the axon terminal, Figure 8B.

### 3.2. Effect of antidromic activation of STN-DBS on neural firing in the cortex

**Figure 9:**
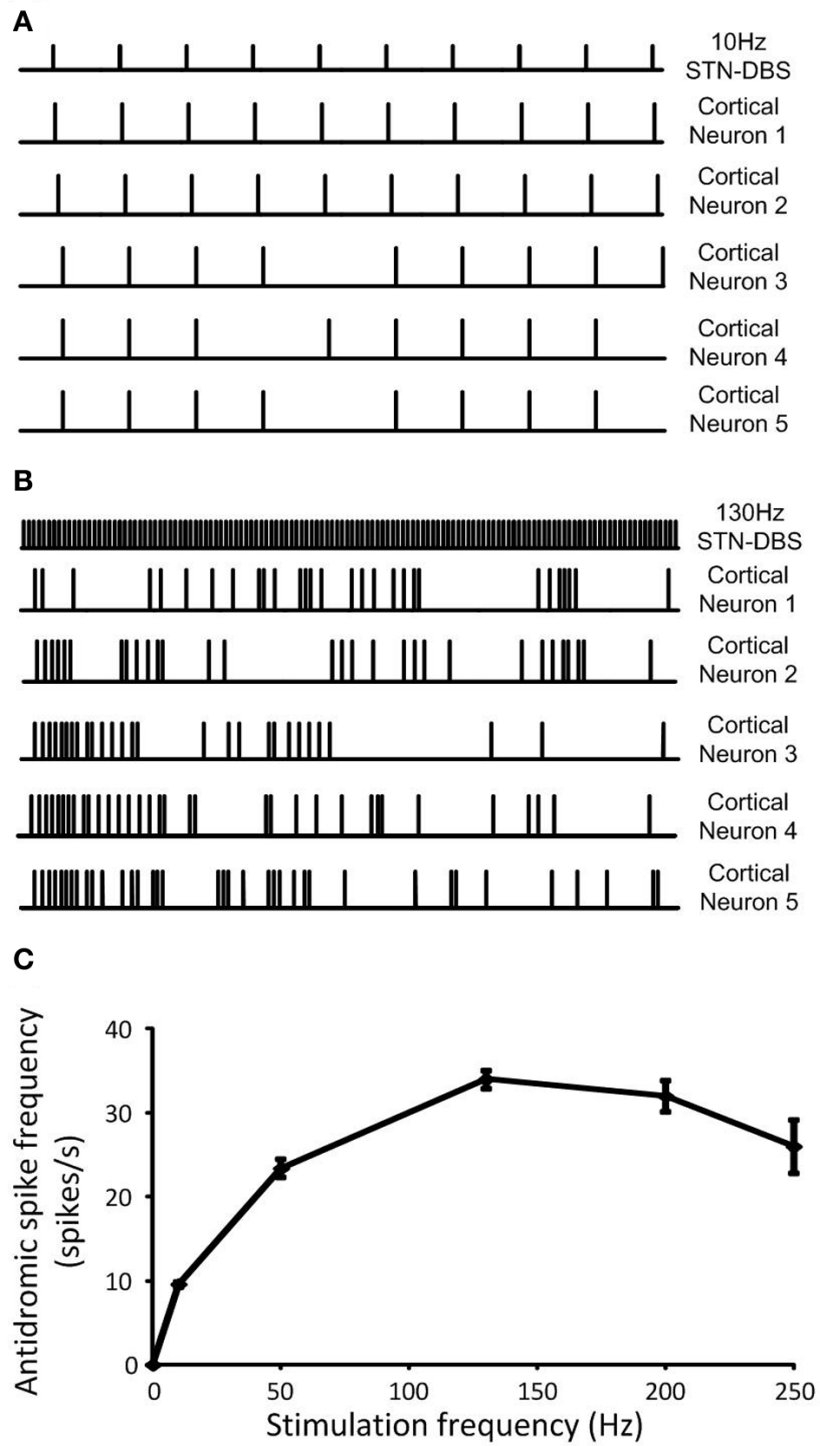
**(A)** Regular neural firing evoked antidromically at the DBS frequency was exhibited in cortical neurons when low frequency DBS was applied while **(B)** random neural firing was generated during high frequency DBS. **(C)** Antidromic spike frequency was increased as DBS frequency increased to a maximum rate at 130 Hz DBS, then, it gradually decreased with DBS frequency. Data shows the mean ± standard deviation of five simulations of duration 1 s each.

At a stimulation frequency of 10 Hz, antidromic cortical excitation was reliably elicited, as over 90% of stimuli were followed by antidromic firing in all cortical neurons, Figure 9A. In contrast, the probability of antidromic firing in response to each stimulation pulse decreased and cortical neurons exhibited highly irregular firing patterns when frequency of the DBS was increased to 130 Hz, Figure 9B. The frequency of antidromic firing increased with DBS frequency to a maximum value of approximately 30 Hz for DBS at 130 Hz, Figure 9C. It then gradually decreased as the DBS frequency was further increased. The probabilities of antidromic firing in response to each DBS stimuli, defined as the number of antidromic spikes over the number of DBS stimulus, were 96.0%, 46.8%, 26.2%, 16.0%, and 10.4% at DBS frequencies of 10, 50, 130, 200, and 250 Hz, respectively. The propagation of regular beta-band oscillatory activity from the cortex to the STN was interrupted as irregular antidromic spikes during DBS replaced dominant beta-band activity in the cortical neurons. Antidromic activation resulted in the suppression of beta-band oscillations in both the cortex and STN effectively, Figures 7, 9.

## 4. Discussion

The aim of this study was to develop a computational model of the cortico-STN-GPe network to examine the effects of antidromic and orthodromic activation on neural firing and beta-band oscillatory activity among cortical and STN neurons during STN-DBS. By providing a method with which to separately stimulate cortical neurons antidromically and STN neurons orthodromically, as well to simultaneously elicit antidromic and orthodromic activation as likely occurs physiologically, the model enables the relative contributions of orthodromic and antidromic activation of STN afferent axons projecting from the cortex to the STN to be explored. The results provide new insights into the possible mechanisms by which STN-DBS modulates and disrupts abnormal neural activity in both the cortex and STN to exert its therapeutic effects.

In this study, based on the findings of the most recent experimental studies, STN afferent axons originating from the cortex were the chosen target for DBS. This is in contrast to previous simulation studies in which DBS was either intracellularly applied to single-compartment models of STN neurons (Rubin and Terman, 2004; Feng et al., 2007; Pirini et al., 2009; Kang and Lowery, 2013) or the electric field in the vicinity of the DBS electrode was simulated using finite element methods and its effect on isolated neurons examined (Grill et al., 2004; McIntyre et al., 2004; Grant and Lowery, 2010; Yousif et al., 2010). Experimental observations in 6-OHDA lesioned rats (Gradinaru et al., 2009) using optogenetic technology have identified afferent fibers to the STN as being a major direct target of DBS. In that study, high frequency DBS applied selectively to STN afferent axons was found to improve motor symptoms of Parkinson's disease as measured by rotational behavior and head position. There is increasing evidence which supports the hypothesis that beta activity in the cortex is transmitted and amplified through the basal ganglia network (Jenkinson and Brown, 2011). Hence, suppression of cortical oscillatory inputs to the basal ganglia through stimulation of cortical afferents to the STN may indeed be a primary target for DBS (Eusebio et al., 2011).

### 4.1. Effects of STN-DBS on suppression of beta-band oscillations in cortical and STN neurons

In the present study, the simulation of beta-band oscillations was based on growing evidence that strong cortical beta inputs to the STN, in the presence of increased STN sensitivity to afferent activity as a result of reduced levels of dopamine, results in the synchronization and propagation of beta band activity throughout the basal ganglia network (Magill et al., 2001; Levy et al., 2002; Brown, 2007; Jenkinson and Brown, 2011). STN beta-band oscillations in the model were thus driven by descending synaptic inputs originating in the cortex.

The firing patterns of the simulated STN neurons, in particular oscillatory neural activity within the beta frequency range, were examined during the application of low and high frequency DBS. High frequency DBS suppressed periodic oscillatory activity in the STN and beta-band activity in both the STN and cortex, while low frequency DBS boosted beta-band activity in the STN through resonant effects. The results of the model are consistent with recent experimental observations in which frequency dependent effects of DBS selectively applied to afferent axons to the STN have been shown both on firing times and bursting frequency in the STN in 6-OHDA lesioned rats (Gradinaru et al., 2009). In addition, recent studies in patients with Parkinson's disease showed attenuation of beta rhythms in both the STN and motor cortex during high frequency STN-DBS in the region where cortico-basal ganglia network axon bundles are located (Whitmer et al., 2012). The deleterious effects of DBS at low frequencies have also been demonstrated in several clinical studies which have quantified tapping performance or the Unified Parkinson's Disease Rating Scale (UPDRS) (Timmermann et al., 2004; Chen et al., 2007; Eusebio et al., 2008). Similar results were also observed in a previous computational model of the closed loop cortico-basal ganglia network which illustrated resonant effects of low frequency DBS on STN beta-band activity when relative low baseline levels of beta-band activity were simulated (Kang and Lowery, 2013).

### 4.2. Effects of antidromic and orthodromic activation of neural firing in the STN during DBS

Action potentials in STN afferent axons projecting from the cortex activated by local DBS propagate in both the antidromic direction toward cell bodies and the orthodromic direction toward axon terminals (Hammond et al., 2008). In this model, the effectiveness of high frequency STN-DBS in suppressing beta-band power in the STN was found to be similar when DBS was applied either antidromically or orthodromically, however, the mechanism by which it exerted its influence was different in both cases. To stimulate antidromic or orthodromic activation in isolation propagation of action evoked by DBS in the unwanted direction was blocked. The combined effect of simulated antidromic and orthodromic activation on the STN afferent axons during DBS resulted in regular firing of STN neurons driven by DBS at the stimulation frequency, Figure 7A. Similar high frequency firing of the STN was also observed when orthodromic activation was simulated in isolation. The model behavior is supported by reported experimental observations. McConnell et al. (2012) measured the coherence between GPe and SNr neurons during high frequency DBS and showed a shift in the frequency at which coherence was observed from the low-frequency band to the stimulation frequency. They suggested that high frequency DBS replaced pathological low-frequency activity with a regularized pattern of neuronal firing at DBS frequency (McConnell et al., 2012), as observed in the model, Figures 5A, 7A.

In contrast, antidromic activation of the STN afferent axons resulted in irregular neural firing with a low firing probability in the STN which, in turn, disturbed beta oscillatory activity in the STN, Figure 7A. Rather than having a direct excitatory effect on the firing times of the STN neurons, in this case the disruption of firing was due to modulation of neural firing in the cortex. Increasing the frequency of DBS resulted in a decrease in the beta-band power in the STN at high DBS frequencies, while DBS at 20 Hz resulted in a small increase in beta-band power, Figure 7B. Oscillatory activity in the STN at the frequency of the applied DBS was increased when compared to the reference condition without DBS, Figure 7C. A similar finding of changes in sum of spectral power over stimulation frequency bands in the GPe and SNr was also observed in experimental data recording in 6-OHDA lesioned rats (McConnell et al., 2012).

When neural firing in the axon collaterals was examined during orthodromic activation alone, action potentials were observed to propagate along the cortical main axon, originating from the cortical soma, without any changes in firing rate or firing pattern. Once they reached the axon collateral, the firing rate and pattern were altered due to the subsequent loss of axonal spikes by DBS, resulting in stimulus-driven neural firing in the latter compartments, Figure 8A. Beta-band power, therefore, decreased in the last axon collateral compartment when compared to the first compartment, Figure 8B.

### 4.3. Effects of antidromic activation of neural firing in the cortex during STN-DBS

When DBS antidromically activated cortical neurons, cortical neurons regularly fired during low frequency DBS at 10 Hz. In contrast, high frequency DBS at 130 Hz resulted in highly irregular neural firing, Figures 9A,B, respectively. Activation of cortical neurons by high frequency DBS was not robust, with the firing probability in response to DBS stimuli reduced when compared to low frequency DBS. The maximum antidromic spike frequency was observed for a DBS frequency of 130 Hz, Figure 9C. The relationship between DBS frequency and firing frequency of cortical neurons in the model, Figure 9C, is in close agreement with the experimental results in 6-OHDA lesioned rats reported by Li et al. (2012) which examined the effects of DBS on the firing probability in corticofugal projection neurons, suppression of beta rhythms and restoration of motor control (see their Figure 2). Their data showed increased burst discharge frequency and highly synchronized beta-band oscillatory activity of cortical neurons. These abnormal neural activities in the cortex were eliminated and replaced with irregular firing pattern by antidromic activation of the cortico-STN pathway during high frequency DBS. Irregular firing and the reduced firing probability of cortical neurons in the model was found to depend on the inhibitory effect of GABAergic cortical interneurons which were also affected by DBS. However, other possible factors may also affect the reliability of cortical activation through antidromic activation during DBS. Chomiak and Hu (2007) suggested that at membrane potentials below −55 mV, cortical neurons showed significant reduction in the probability of antidromic invasion. They also demonstrated that poorly myelinated axons failed to transmit action potentials at high stimulation frequencies resulting in low frequency spike output.

Although the model provides a close agreement with a range of available experimental data, as with any computational model there are limitations which should be considered. As the cortical neurons were simulated as multi-compartment model, while other neurons are single-compartment model, there is a slight delay as action potentials propagate from the cortex to the STN which is not present, for example for the input from the GPe to the STN. The model of the effect of antidromic activation of DBS, Figure 4A does not capture cancelation of action potentials within the axon collateral. However, due to the high frequency nature of the stimulation, most action potential cancelation occurs in the main axon and very few action potentials manage to reach the axon collateral, resulting in a negligible effect on the results. The model presented in this study focuses on the behavior of STN and cortical neurons as a result of antidromic and orthodromic effects of DBS and does not fully incorporate the closed loop cortico-basal ganglia network. The number of neurons present in each nucleus is lower than that what would be physiologically realistic. However, preliminary simulation studies using the model indicated that similar results are expected if the number of neurons was increased. The extracellular potential generated by DBS has been simplified and was represented by a means of point current source rather than a geometrically realistic model of a DBS electrode. It has been shown that the point source is a valid approximation for application of monopolar DBS when predicting activation thresholds of model neurons located within 3 mm of the electrode (Zhang and Grill, 2010). However, effects due to the presence of the electrode shaft and encapsulation tissue are not captured and the value of the applied voltage for neurons very close to the electrode is may be different than it would be in models that include these effects. Interneurons were modeled using a simple spiking type model. Nevertheless, the model facilitates an exploration of the relative contribution of effects of antidromic and orthodromic activation of neural firing in cortical neurons and STN neurons which would not be possible *in vivo* or *in vitro* and exhibited a range of results which are consistent with available experimental data. In future studies, the model presented may be coupled to more detailed volume conductor models that capture the effects of electrode geometry, variations in tissue properties and electrode configuration. The model could then be used to investigate effects of STN-DBS at different contact locations. The model could also be extended to simulate the closed loop thalamocortical-basal gangia network and the effect of DBS on neural activity associated with parkinsonian tremor.

## 5. Conclusion

The results of a new computational model presented here, indicate that high frequency DBS applied to STN afferents originating from the cortex modulates the behavior of both cortical and STN neurons, resulting in suppression of oscillatory beta-band neural activity in parkinsonian conditions. The model exhibited overlapping effects of antidromic and orthodromic activation during DBS, with each also resulting in suppressing beta band activity when applied in isolation. Orthodromic activation of STN afferents resulted in firing of STN neurons in synchrony with the DBS stimulus, thereby suppressing the propagation of beta activity throughout the network. Antidromic activation similarly resulted in suppression of beta-band activity in both the cortex and STN. However, this was mediated through the alternative mechanism of disruption of cortical firing, thereby disrupting propagation of beta activity from the cortex to the STN. The results thus indicate that STN-DBS can effectively suppress pathological oscillations of STN and cortical neurons either by antidromic or orthodromic activation of cortical afferents, and that the efficacies of both mechanisms are comparable. The results further suggest that in the physiological situation, DBS may do both i.e., it may orthodromically activate STN fibers while simultaneously suppressing oscillatory activity in the cortex through antidromic stimulation. The model behavior agrees closely with experimental recordings in both the cortex and STN in previous animal studies and presents a possible mechanism of action of STN-DBS in Parkinson's disease by which pathological oscillations are attenuated through a combination of orthodromic and antidromic activation. The results of the present study support the suggestion that cortical neurons may indeed be one of main targets of DBS and that similar therapeutic effects could potentially be elicited through the stimulation of cortical neurons either directly or through alternative pathways.

### 5.1. Data sharing

Frontiers supports the policy of data sharing, and authors are advised to make freely available any materials and information described in their article, and any data relevant to the article (while not compromising confidentiality in the context of human-subject research) that may be reasonably requested by others for the purpose of academic and non-commercial research. In regards to deposition of data and data sharing through databases, Frontiers urges authors to comply with the current best practices within their discipline.

## Funding

This work was supported by Irish Research Council for Science, Engineering and Technology Grant RS/2009/1330.

### Conflict of interest statement

The authors declare that the research was conducted in the absence of any commercial or financial relationships that could be construed as a potential conflict of interest.

## References

[B1] Albin R. L. Young A. B. Penney J. B. (1989). The functional anatomy of basal ganglia disorders. Trends Neurosci. 12, 366–375 10.1016/0166-2236(89)90074-X 2479133

[B2] Ashby P. Paradiso G. Saint-Cyr J. A. Chen R. Lang A. E. Lozano A. M. (2001). Potentials recorded at the scalp by stimulation near the human subthalamic nucleus. Clin. Neurophysiol. 112, 431–437 10.1016/S1388-2457(00)00532-0 11222963

[B3] Baker K. B. Montgomery E. B. Jr. Rezai A. R. Burgess R. Lüders H. O. (2002). Subthalamic nucleus deep brain stimulus evoked potentials: physiological and therapeutic implications. Mov. Disord. 17, 969–983 10.1002/mds.10206 12360546

[B4] Baufreton J. Atherton J. F. Surmeier D. J. Bevan M. D. (2005). Enhancement of excitatory synaptic integration by GABAergic inhibition in the subthalamic nucleus. J. Neurosci. 25, 8505–8517 10.1523/JNEUROSCI.1163-05.2005 16162932 PMC6725678

[B5] Bergman H. Wichmann T. Karmon B. DeLong M. R. (1994). The primate subthalamic nucleus. II. Neuronal activity in the MPTP model of parkinsonism. J. Neurophysiol. 72, 507–520 7983515 10.1152/jn.1994.72.2.507

[B6] Beurrier C. Congar P. Bioulac B. Hammond C. (1999). Subthalamic nucleus neurons switch from single-spike activity to burst-firing mode. J. Neurosci. 19, 599–609 9880580 10.1523/JNEUROSCI.19-02-00599.1999 PMC6782207

[B7] Bronte-Stewart H. Barberini C. Koop M. M. Hill B. C. Henderson J. M. Wingeier B. (2009). The STN beta-band profile in Parkinson's disease is stationary and shows prolonged attenuation after deep brain stimulation. Exp. Neurol. 215, 20–28 10.1016/j.expneurol.2008.09.008 18929561

[B8] Brown P. (2007). Abnormal oscillatory synchronisation in the motor system leads to impaired movement. Curr. Opin. Neurobiol. 17, 656–664 10.1016/j.conb.2007.12.001 18221864

[B9] Brown P. Oliviero A. Mazzone P. Insola A. Tonali P. Lazzaro V. D. (2001). Dopamine dependency of oscillations between subthalamic nucleus and pallidum in Parkinson's disease. J. Neurosci. 21, 1033–1038 11157088 10.1523/JNEUROSCI.21-03-01033.2001 PMC6762327

[B10] Brown P. Williams D. (2005). Basal ganglia local field potential activity: character and functional significance in the human. Clin. Neurophysiol. 116, 2510–2519 10.1016/j.clinph.2005.05.009 16029963

[B11] Canteras N. S. Shammah-Lagnado S. J. Silva B. A. Ricardo J. A. (1990). Afferent connections of the subthalamic nucleus: a combined retrograde and anterograde horseradish peroxidase study in the rat. Brain Res. 513, 43–59 10.1016/0006-8993(90)91087-W 2350684

[B12] Chen C. C. Litvak V. Gilbertson T. Kühn A. Lu C. S. Lee S. T. (2007). Excessive synchronization of basal ganglia neurons at 20 Hz slows movement in Parkinson's disease. Exp. Neurol. 205, 214–221 10.1016/j.expneurol.2007.01.027 17335810

[B13] Chomiak T. Hu B. (2007). Axonal and somatic filtering of antidromically evoked cortical excitation by simulated deep brain stimulation in rat brain. J. Physiol. 579, 403–412 10.1113/jphysiol.2006.124057 17170044 PMC2075404

[B14] Cruz A. V. Mallet N. Magill P. J. Brown P. Averbeck B. B. (2009). Effects of dopamine depletion on network entropy in the external globus pallidus. J. Neurophysiol. 102, 1092–1102 10.1152/jn.00344.2009 19535481 PMC2724349

[B15] Dejean C. Hyland B. Arbuthnott G. (2009). Cortical effects of subthalamic stimulation correlate with behavioral recovery from dopamine antagonist induced akinesia. Cereb. Cortex 19, 1055–1063 10.1093/cercor/bhn149 18787234

[B16] Eusebio A. Chen C. C. Lu C. S. Lee S. T. Tsai C. H. Limousin P. (2008). Effects of low-frequency stimulation of the subthalamic nucleus on movement in Parkinson's disease. Exp. Neurol. 209, 125–130 10.1016/j.expneurol.2007.09.007 17950279 PMC2288636

[B17] Eusebio A. Thevathasan W. Doyle Gaynor L. Pogosyan A. Bye E. Foltynie T. (2011). Deep brain stimulation can suppress pathological synchronisation in parkinsonian patients. J. Neurol. Neurosurg. Psychiatry 82, 569–573 10.1136/jnnp.2010.217489 20935326 PMC3072048

[B18] Feng X.-J. Shea-Brown E. Greenwald B. Kosut R. Rabitz H. (2007). Optimal deep brain stimulation of the subthalamic nucleus–a computational study. J. Comput. Neurosci. 23, 265–282 10.1007/s10827-007-0031-0 17484043

[B19] Foust A. J. Yu Y. Popovic M. Zecevic D. McCormick D. A. (2011). Somatic membrane potential and kv1 channels control spike repolarization in cortical axon collaterals and presynaptic boutons. J. Neurosci. 31, 15490–15498 10.1523/JNEUROSCI.2752-11.2011 22031895 PMC3225031

[B20] Fraix V. Pollak P. Vercueil L. Benabid A.-L. Mauguière F. (2008). Effects of subthalamic nucleus stimulation on motor cortex excitability in parkinson's disease. Clin. Neurophysiol. 119, 2513–2518 10.1016/j.clinph.2008.07.217 18783985

[B21] Fujimoto K. Kita H. (1993). Response characteristics of subthalamic neurons to the stimulation of the sensorimotor cortex in the rat. Brain Res. 609, 185–192 10.1016/0006-8993(93)90872-K 8508302

[B22] Gradinaru V. Mogri M. Thompson K. R. Henderson J. M. Deisseroth K. (2009). Optical deconstruction of parkinsonian neural circuitry. Science 324, 354–359 10.1126/science.1167093 19299587 PMC6744370

[B23] Grant P. F. Lowery M. M. (2010). Effect of dispersive conductivity and permittivity in volume conductor models of deep brain stimulation. IEEE Trans. Biomed. Eng. 57, 2386–2393 10.1109/TBME.2010.2055054 20595081

[B24] Grill W. M. Cantrell M. B. Robertson M. S. (2008). Antidromic propagation of action potentials in branched axons: implications for the mechanisms of action of deep brain stimulation. J. Comput. Neurosci. 24, 81–93 10.1007/s10827-007-0043-9 17562157

[B25] Grill W. M. Snyder A. N. Miocinovic S. (2004). Deep brain stimulation creates an informational lesion of the stimulated nucleus. Neuroreport 15, 1137–1140 10.1097/00001756-200405190-00011 15129161

[B26] Hahn P. J. McIntyre C. C. (2010). Modeling shifts in the rate and pattern of subthalamopallidal network activity during deep brain stimulation. J. Comput. Neurosci. 28, 425–441 10.1007/s10827-010-0225-8 20309620 PMC2881193

[B27] Hammond C. Ammari R. Bioulac B. Garcia L. (2008). Latest view on the mechanism of action of deep brain stimulation. Mov. Disord. 23, 2111–2121 10.1002/mds.22120 18785230

[B28] Hanson J. E. Smith Y. Jaeger D. (2004). Sodium channels and dendritic spike initiation at excitatory synapses in globus pallidus neurons. J. Neurosci. 24, 329–340 10.1523/JNEUROSCI.3937-03.2004 14724231 PMC6729996

[B29] Holgado A. J. N. Terry J. R. Bogacz R. (2010). Conditions for the generation of beta oscillations in the subthalamic nucleus-globus pallidus network. J. Neurosci. 30, 12340–12352 10.1523/JNEUROSCI.0817-10.2010 20844130 PMC6633459

[B30] Izhikevich E. M. (2003). Simple model of spiking neurons. IEEE Trans. Neural Netw. 14, 1569–1572 10.1109/TNN.2003.820440 18244602

[B31] Jaeger D. (2003). The control of spiking by synaptic input in striatal and pallidal neurons, in The Basal Ganglia VI - Advances in Behavioral Biology, Vol. 54, eds Graybiel A. M. Delong M. R. Kitai S. T. (New York, NY: Kluwer Academic Press), 209–216 10.1007/978-1-4615-0179-4_21

[B32] Jenkinson N. Brown P. (2011). New insights into the relationship between dopamine, beta oscillations and motor function. Trends Neurosci. 34, 611–618 10.1016/j.tins.2011.09.003 22018805

[B33] Kang G. Lowery M. M. (2009). A model of pathological oscillations in the basal ganglia and deep brain stimulation in Parkinson's disease. Conf. Proc. IEEE Eng. Med. Biol. Soc. 2009, 3909–3912 10.1109/IEMBS.2009.5333557 19964318

[B34] Kang G. Lowery M. M. (2013). Interaction of oscillations, and their suppression via deep brain stimulation, in a model of the cortico-basal ganglia network. IEEE Trans. Neural Syst. Rehabil. Eng. 21, 244–253 10.1109/TNSRE.2013.2241791 23476006

[B35] Kita H. Kitai S. T. (1994). The morphology of globus pallidus projection neurons in the rat: an intracellular staining study. Brain Res. 636, 308–319 10.1016/0006-8993(94)91030-8 8012814

[B36] Kita T. Kita H. (2012). The subthalamic nucleus is one of multiple innervation sites for long-range corticofugal axons: a single-axon tracing study in the rat. J. Neurosci. 32, 5990–5999 10.1523/JNEUROSCI.5717-11.2012 22539859 PMC3479642

[B37] Kühn A. A. Kempf F. Brücke C. Gaynor Doyle L. Martinez-Torres I. Pogosyan A. (2008). High-frequency stimulation of the subthalamic nucleus suppresses oscillatory beta activity in patients with parkinson's disease in parallel with improvement in motor performance. J. Neurosci. 28, 6165–6173 10.1523/JNEUROSCI.0282-08.2008 18550758 PMC6670522

[B38] Kühn A. A. Kupsch A. Schneider G.-H. Brown P. (2006). Reduction in subthalamic 8-35 Hz oscillatory activity correlates with clinical improvement in Parkinson's disease. Eur. J. Neurosci. 23, 1956–1960 10.1111/j.1460-9568.2006.04717.x 16623853

[B39] Kühn A. A. Trottenberg T. Kivi A. Kupsch A. Schneider G.-H. Brown P. (2005). The relationship between local field potential and neuronal discharge in the subthalamic nucleus of patients with Parkinson's disease. Exp. Neurol. 194, 212–220 10.1016/j.expneurol.2005.02.010 15899258

[B40] Levy R. Ashby P. Hutchison W. D. Lang A. E. Lozano A. M. Dostrovsky J. O. (2002). Dependence of subthalamic nucleus oscillations on movement and dopamine in Parkinson's disease. Brain 125, 1196–1209 10.1093/brain/awf128 12023310

[B41] Li Q. Ke Y. Chan D. C. W. Qian Z.-M. Yung K. K. L. Ko H. (2012). Therapeutic deep brain stimulation in parkinsonian rats directly influences motor cortex. Neuron 76, 1030–1041 10.1016/j.neuron.2012.09.032 23217750

[B42] Li S. Arbuthnott G. W. Jutras M. J. Goldberg J. A. Jaeger D. (2007). Resonant antidromic cortical circuit activation as a consequence of high-frequency subthalamic deep-brain stimulation. J. Neurophysiol. 98, 3525–3537 10.1152/jn.00808.2007 17928554

[B43] Litvak V. Jha A. Eusebio A. Oostenveld R. Foltynie T. Limousin P. (2011). Resting oscillatory cortico-subthalamic connectivity in patients with parkinson's disease. Brain 134, 359–374 10.1093/brain/awq332 21147836

[B44] MacKinnon C. D. Webb R. M. Silberstein P. Tisch S. Asselman P. Limousin P. (2005). Stimulation through electrodes implanted near the subthalamic nucleus activates projections to motor areas of cerebral cortex in patients with parkinson's disease. Eur. J. Neurosci. 21, 1394–1402 10.1111/j.1460-9568.2005.03952.x 15813949

[B45] Magill P. J. Bolam J. P. Bevan M. D. (2001). Dopamine regulates the impact of the cerebral cortex on the subthalamic nucleus-globus pallidus network. Neuroscience 106, 313–330 10.1016/S0306-4522(01)00281-0 11566503

[B46] McCarthy M. M. Brown E. N. Kopell N. (2008). Potential network mechanisms mediating electroencephalographic beta rhythm changes during propofol-induced paradoxical excitation. J. Neurosci. 28, 13488–13504 10.1523/JNEUROSCI.3536-08.2008 19074022 PMC2717965

[B47] McConnell G. C. So R. Q. Hilliard J. D. Lopomo P. Grill W. M. (2012). Effective deep brain stimulation suppresses low-frequency network oscillations in the basal ganglia by regularizing neural firing patterns. J. Neurosci. 32, 15657–15668 10.1523/JNEUROSCI.2824-12.2012 23136407 PMC3502634

[B48] McIntyre C. C. Grill W. M. Sherman D. L. Thakor N. V. (2004). Cellular effects of deep brain stimulation: model-based analysis of activation and inhibition. J. Neurophysiol. 91, 1457–1469 10.1152/jn.00989.2003 14668299

[B49] McIntyre C. C. Hahn P. J. (2010). Network perspectives on the mechanisms of deep brain stimulation. Neurobiol. Dis. 38, 329–337 10.1016/j.nbd.2009.09.022 19804831 PMC2862840

[B50] Miocinovic S. Parent M. Butson C. R. Hahn P. J. Russo G. S. Vitek J. L. (2006). Computational analysis of subthalamic nucleus and lenticular fasciculus activation during therapeutic deep brain stimulation. J. Neurophysiol. 96, 1569–1580 10.1152/jn.00305.2006 16738214

[B51] Nakanishi H. Kita H. Kitai S. T. (1987). Intracellular study of rat substantia nigra pars reticulata neurons in an *in vitro* slice preparation: electrical membrane properties and response characteristics to subthalamic stimulation. Brain Res. 437, 45–55 10.1016/0006-8993(87)91525-3 3427482

[B52] Otsuka T. Abe T. Tsukagawa T. Song W.-J. (2004). Conductance-based model of the voltage-dependent generation of a plateau potential in subthalamic neurons. J. Neurophysiol. 92, 255–264 10.1152/jn.00508.2003 15212440

[B53] Pirini M. Rocchi L. Sensi M. Chiari L. (2009). A computational modelling approach to investigate different targets in deep brain stimulation for Parkinson's disease. J. Comput. Neurosci. 26, 91–107 10.1007/s10827-008-0100-z 18553128

[B54] Plonsey R. Heppner D. B. (1967). Considerations of quasi-stationarity in electrophysiological systems. Bull. Math. Biophys. 29, 657–664 10.1007/BF02476917 5582145

[B55] Pospischil M. Toledo-Rodriguez M. Monier C. Piwkowska Z. Bal T. Frénac Y. (2008). Minimal hodgkin-huxley type models for different classes of cortical and thalamic neurons. Biol. Cybern. 99, 427–441 10.1007/s00422-008-0263-8 19011929

[B56] Rattay F. (1986). Analysis of models for external stimulation of axons. IEEE Trans. Biomed. Eng. 33, 974–977 10.1109/TBME.1986.325670 3770787

[B57] Raz A. Feingold A. Zelanskaya V. Vaadia E. Bergman H. (1996). Neuronal synchronization of tonically active neurons in the striatum of normal and parkinsonian primates. J. Neurophysiol. 76, 2083–2088 8890317 10.1152/jn.1996.76.3.2083

[B58] Rubin J. E. Terman D. (2004). High frequency stimulation of the subthalamic nucleus eliminates pathological thalamic rhythmicity in a computational model. J. Comput. Neurosci. 16, 211–235 10.1023/B:JCNS.0000025686.47117.67 15114047

[B59] Santaniello S. Montgomery E. B. Jr. Gale J. T. Sarma S. V. (2012). Non-stationary discharge patterns in motor cortex under subthalamic nucleus deep brain stimulation. Front. Integr. Neurosci. 6:35 10.3389/fnint.2012.00035 22754509 PMC3385519

[B60] Sharott A. Magill P. J. Bolam J. P. Brown P. (2005a). Directional analysis of coherent oscillatory field potentials in the cerebral cortex and basal ganglia of the rat. J. Physiol. 562, 951–963 10.1113/jphysiol.2004.073189 15550466 PMC1665537

[B61] Sharott A. Magill P. J. Harnack D. Kupsch A. Meissner W. Brown P. (2005b). Dopamine depletion increases the power and coherence of beta-oscillations in the cerebral cortex and subthalamic nucleus of the awake rat. Eur. J. Neurosci. 21, 1413–1422 10.1111/j.1460-9568.2005.03973.x 15813951

[B62] Sims R. E. Woodhall G. L. Wilson C. L. Stanford I. M. (2008). Functional characterization of gabaergic pallidopallidal and striatopallidal synapses in the rat globus pallidus *in vitro*. Eur J. Neurosci. 28, 2401–2408 10.1111/j.1460-9568.2008.06546.x 19087170

[B63] Smith Y. Hazrati L. N. Parent A. (1990). Efferent projections of the subthalamic nucleus in the squirrel monkey as studied by the pha-l anterograde tracing method. J. Comp. Neurol. 294, 306–323 10.1002/cne.902940213 2332533

[B64] Tass P. Smirnov D. Karavaev A. Barnikol U. Barnikol T. Adamchic I. (2010). The causal relationship between subcortical local field potential oscillations and parkinsonian resting tremor. J. Neural Eng. 7:16009 10.1088/1741-2560/7/1/016009 20083863

[B65] Terman D. Rubin J. E. Yew A. C. Wilson C. J. (2002). Activity patterns in a model for the subthalamopallidal network of the basal ganglia. J. Neurosci. 22, 2963–2976 11923461 10.1523/JNEUROSCI.22-07-02963.2002 PMC6758326

[B66] Timmermann L. Wojtecki L. Gross J. Lehrke R. Voges J. Maarouf M. (2004). Ten-Hertz stimulation of subthalamic nucleus deteriorates motor symptoms in Parkinson's disease. Mov. Disord. 19, 1328–1333 10.1002/mds.20198 15389990

[B67] Whitmer D. de Solages C. Hill B. Yu H. Henderson J. M. Bronte-Stewart H. (2012). High frequency deep brain stimulation attenuates subthalamic and cortical rhythms in parkinson's disease. Front. Hum. Neurosci. 6:155 10.3389/fnhum.2012.00155 22675296 PMC3366347

[B68] Yousif N. Purswani N. Bayford R. Nandi D. Bain P. Liu X. (2010). Evaluating the impact of the deep brain stimulation induced electric field on subthalamic neurons: a computational modelling study. J. Neurosci. Methods 188, 105–112 10.1016/j.jneumeth.2010.01.026 20116398

[B69] Yu Y. Shu Y. McCormick D. A. (2008). Cortical action potential backpropagation explains spike threshold variability and rapid-onset kinetics. J. Neurosci. 28, 7260–7272 10.1523/JNEUROSCI.1613-08.2008 18632930 PMC2664555

[B70] Zhang T. C. Grill W. M. (2010). Modeling deep brain stimulation: point source approximation versus realistic representation of the electrode. J. Neural Eng. 7:066009 10.1088/1741-2560/7/6/066009 21084730 PMC3076020

